# How do Adolescents’ Perceptions of Relationships with Teachers Change during Upper-Secondary School Years?

**DOI:** 10.1007/s10964-019-01155-3

**Published:** 2019-11-02

**Authors:** Sevgi Bayram Özdemir, Metin Özdemir

**Affiliations:** School of Law, Psychology and Social Work, Örebro University, 701 82 Örebro, Sweden

**Keywords:** Teacher-student relationship, School satisfaction, Teacher fairness, Teacher support

## Abstract

The student-teacher relationship has mostly been assumed to be static. This approach is limited in providing information on how relationships with teachers evolve over time, and how possible changes affect young people’s adjustment. To address this gap in knowledge, the present study examined whether adolescents follow different trajectories in their perceptions of relationship with teachers and whether students on different trajectories differ from each other in their adjustment. The sample included 829 students residing in Sweden (*M*_*age*_ = 13.43, *SD* = 0.55, 51% girls). Three distinct teacher-relationship trajectories were identified. More than half (66%) of the adolescents (*average-stable trajectory*) reported an average level of positive relationships with teachers at grade 7, and did not change significantly over the three years. About 24% of the adolescents (*high-increasing trajectory*) reported a high level of fair and supportive teacher-relationships at T1, and continued to increase in their positive views from grade 7 to grade 9. Ten percent of the adolescents (*average-declining trajectory*) reported an average level of positive relationships with teachers at grade 7, but showed a decline in their positive views towards teachers over time. Relative to adolescents on an average-stable trajectory, adolescents on a high-increasing trajectory reported greater school satisfaction, higher achievement values, and lower failure anticipation. By contrast, adolescents in the average-declining group reported worsening school adjustment. No significant moderating effects of immigrant status and gender were found. These findings highlight the importance of the association between the continuous experience of supportive and fair teacher treatment and youth adjustment.

## Introduction

Children spend a significant amount of their time in school. Their experiences of teachers have implications for their well-being and school functioning. A growing body of literature shows that supportive relationships with teachers foster students’ engagement in learning activities (see Roorda et al. 2011 for a meta-analysis), feelings of school belonging (Wang and Eccles 2012), and academic skills (Maldonado-Carreno and Votruba-Drzal 2011). Such positive relationships also protect children from developing emotional and behavioral problems (see Lei et al. 2016 for a meta-analysis). Despite an increasing interest in the role of student-teacher relationships in children’s adjustment, the current literature has two major limitations.

First, a majority of the studies have focused on early childhood or the childhood period (for a few exceptions, see, for example, Longobardi et al. 2016; Reddy et al. 2003), and do not provide insight into how the quality of the student-teacher relationship is related to young people’s adjustment during the upper-secondary school years (ages 13–15). During this developmental period, adolescents experience pubertal changes, and often undergo shifts in their sense of self. At the same time, they are situated in a school context where there is a growing emphasis on standardized testing, and relatedly, a greater demand for academic performance. These changes and challenges may put young people at risk of experiencing academic and social adjustment problems. Teachers might be particularly important during this developmental period as they can provide a safe context for youth by providing support and guidance, by transmitting perspectives, and by helping them negotiate their way through the challenges.

Second, most studies (except for a few, e.g., Bosman et al. 2018; O’Connor and McCartney 2007; Spilt et al. 2012) have examined the student-teacher relationship at a single point in time, and predicted its effect on subsequent developmental outcomes. This approach assumes that the student-teacher relationship is static, and does not consider how relationship quality changes over time. As highlighted in the interpersonal-relationship literature, sustained or increasing exposure to interpersonal stressors may have a greater and longer lasting impact on adolescents’ development than experiences of temporary difficulties at a certain moment in time (e.g., Lester et al. 2013). In a similar way, the continuous experience of a supportive relationship may be more favorable for adolescents. To address these important gaps in the literature, the present study examined whether adolescents who follow different trajectories in their relationship with teachers differ from each other in their school adjustment.

### Supportive and Fair Relationships with Teachers: Theoretical Framework

Different theoretical arguments have been used to explain why student-teacher relationships may play a role in the development and adjustment of children. The first prevailing theoretical argument is based on the attachment theory (Bowlby 1969). This theoretical perspective is mostly adopted in studies focusing on the preschool and elementary school years, and highlights the view that close relationships with teachers may create a secure base for children, and in turn, foster children’s positive sense of self, willingness to explore, and internal motivation to learn new things. By contrast, poor relationships with teachers, largely defined in terms of high conflict and low closeness, may lead children to feel insecure and distressed in the school environment, and thus hamper their adjustment and functioning in school (see Verschueren and Koomen 2012; Wentzel 2016 for an overview).

The second theoretical argument is built on the premises of social-motivation theories (such as Self Determination Theory, Deci and Ryan 2000), and emphasizes that young people’s basic psychological needs, including a sense of belonging or relatedness, need to be satisfied within the school context in order for them to engage positively in social and academic tasks in the classroom. A supportive teacher-child relationship may convey to children that they are valued at school, and foster the children’s sense of belonging. In turn, the feelings engendered by the relationship may boost their positive feelings and thoughts about school and help them acquire better academic skills (see Roorda et al. 2011 for an overview; Roeser et al. 1996).

The final theoretical argument has a social support perspective, and postulates that support from teachers is a personal resource that can act as a buffer against the challenges students may face (see Wentzel 2016 for an overview). This perspective considers multiple dimensions of support, including *informational* (i.e., the extent to which students perceive that they are given clear guidance on what is expected and valued in the classroom), *instrumental* (i.e., the extent to which students get necessary help and advice from teachers), and *emotional support* (i.e., the extent to which students regard their relationships with teachers as nurturing and emotionally supportive) (Wentzel 2004). It indicates that when students receive necessary teacher support, they tend to be better at dealing with challenges, and in turn, have better social and academic adjustment (Wentzel et al. 2010; see Wentzel 2016 for an overview). Together, despite differences in focus, these three theoretical perspectives complement each other and highlight the importance of secure, close, and supportive relationships with teachers for the adjustment of children and adolescents in a school setting.

In addition to these three prevailing theoretical arguments, a growing body of research emphasizes the importance of a sense of justice and fairness in student-teacher relationships (e.g., Chen and Cui 2019; Gini et al. 2018). These studies use equity theory (Adams 1965) as a conceptual base, and argue that young people become sensitive to social comparisons during adolescence, and may be attentive to how fairly they are treated by their teachers compared to their peers. When students perceive their teachers as fair, the experience may enhance their sense of identity and self-worth (Tyler and Smith 1999), and also their sense of school belonging and connection (Molinari et al. 2013). By contrast, a perception of injustice or a sense of unfairness may put adolescents at risk of psychosomatic symptoms (Lenzi et al. 2013), dissatisfaction and distress (e.g., Gini et al. 2018), and in turn, school adjustment difficulties (Chen and Cui 2019; Ripski and Gregory 2009). Specifically, a recent large-scale study (Chen and Cui 2019) showed that perceived teacher unfairness was negatively associated with achievement in science, with a modest effect size across 52 different countries. Together, these studies indicate that a sense of justice and fairness is one of the fundamental needs of adolescents, which needs to be satisfied in order for adolescents to function well in school. The present study focused on both fairness and support aspects of the perceived teacher relationship, and examined how adolescents’ perceptions change during middle school years, and how such change is linked to their school satisfaction, achievement values, and failure anticipation.

### The Longitudinal Association between Student-Teacher Relationships and Children’s Adjustment

Studies examining the long-term effects of student-teacher relationship quality largely focus on children’s experiences of teachers during preschool, and examine how these early experiences impact the academic, emotional, and behavioral adjustment of children by the time they are in primary school. These studies show that children with poor relationships with their teachers (conceptualized as high conflict and dependency as rated by teachers) during preschool are more likely to show poor academic performance (Hamre and Pianta 2001), to display poor social skills, such as low cooperation and low self-regulatory behaviors, and to engage in externalizing misbehaviors to a greater extent in primary school (Pianta and Stuhlman 2004). By contrast, children who have had a close relationship with their teachers during preschool (based on teacher ratings of closeness) are more likely to have better language and cognitive skills (Peisner‐Feinberg et al. 2001), and to engage in social behaviors (e.g., initiating and sharing friendships) (Berry and O’Connor 2010). Together, and in support of the premises of attachment theory, these studies suggest that positive early experiences with teachers may provide children with a secure emotional base, and predict their academic and social functioning as they move into a formal educational setting.

From childhood and onwards, the nature of the student-teacher relationship changes. Teachers increasingly become instructors rather than caregivers. Students also have fewer opportunities to interact with their teachers one-on-one. In line with these changes, a decline in the quality of relationships with teachers from childhood to early adolescence has been demonstrated (e.g., Hughes and Cao 2018; O'Connor 2010). For example, in a large-scale longitudinal study, O'Connor (2010) followed children from 1st to 5th grade, and reported that, on average, the quality of the teacher-child relationship (operationalized as “high close” and “low conflict” based on teacher ratings) declined slightly through elementary school. Despite changes in the nature of the teacher-student relationship, recent studies suggest that the quality of the relationship still plays a crucial role in the adjustment of children up until the middle school years. For example, improvement in the teacher-student relationship (measured as an increase in the teacher rating of closeness) was found to be associated with an increase in reading skills (McCormick and O'Connor 2015), the display of fewer psychosocial problems, such as depressive symptoms and socially withdrawn behaviors (Maldonado-Carreno and Votruba-Drzal 2011), and less engagement in rule-breaking and aggressive behaviors (Maldonado-Carreno and Votruba-Drzal 2011; Rudasill et al. 2010). Together, these findings suggest that, when students form good relationships with their teachers, such relationships may boost their academic commitment and well-being, and protect them from engaging in risky behaviors during childhood.

A majority of the studies that examine the roles of the student-teacher relationship during adolescence are cross-sectional by nature (e.g., Gini et al. 2018; Roeser et al. 1996). However, efforts have been made recently to identify the long-term effects of the quality of the student-teacher relationship on young people’s adjustment during adolescence (e.g., Reddy et al. 2003; Wang and Holcombe 2010). Unlike the studies that focus on early and later childhood, these longitudinal studies do not rely on teacher reports, but examine the extent to which adolescents themselves perceive their teachers as caring, supportive, and fair. It has been shown that when adolescents perceive their teachers as caring and supportive, they are more likely to be satisfied with and interested in school (Wang and Holcombe 2010), to have a sense of belonging in the school environment (Wang and Holcombe 2010), to value learning (Roeser et al. 1998), to engage in class activities (Engels et al. 2016), and in turn, to display better academic performance over time (e.g., Roeser et al. 1998; Wang and Holcombe 2010). Positive relationships with teachers (measured in terms of students’ perceptions of having a close relation to or feeling cared about by their teachers) have also been found to protect adolescents from developing depressive symptoms (Joyce and Early 2014; Reddy et al. 2003), and from being involved in delinquent and violent behaviors over time (Wang et al. 2013). In sum, these studies indicate that relationships with teachers continue to play an essential role in the school and psychosocial adjustment of young people during adolescence.

One of the major limitations of studies of adolescents, however, is that they tend to treat the student-teacher relationship as static (except for a few: e.g., Reddy et al. 2003), and try to predict how the relationship with teachers during early adolescence is linked to young people’s later adjustment outcomes. However, recent studies that focus on childhood have shown that children follow different trajectories in their relationships with teachers, and that the trajectory they actually follow is linked to their long-term adjustment rather than their initial relationship with teachers. For example, O’Connor and McCartney (2007) followed children from preschool to 3rd grade, and identified subgroups of children who followed different growth trajectories. They showed that the children who were rated as having a poor relationship with teachers to start with *and* showed a decline over time were less likely to engage in classroom activities and displayed poorer academic performance than those who had an increasingly good relationship with teachers over time. Similarly, in a recent study, Bosman and colleagues (2018), when examining whether children from preschool to 6th grade followed different trajectories regarding teacher dependency (measured on the basis of teacher ratings), identified two groups in terms of dependency over time: (1) low-decreasing and (2) low-increasing. They showed that students with low-increasing dependency had more problems at 6th grade, including lower levels of vocabulary, technical reading, reading comprehension, math achievement, self-efficacy, and task motivation, than those on a low-decreasing trajectory. Together, these findings highlight the importance of treating the teacher-student relationship as a time-varying construct so as better to understand its associations with young people’s development. However, no study has examined growth trajectories in student-teacher relationships during adolescence, particularly from the adolescents’ own perspectives.

To address this important gap in knowledge, the present study examined (1) whether young people follow different trajectories in their perceived relationships with teachers during upper-secondary school years (from age 13 to 15), and (2) whether students on different teacher-relationship trajectories differ from each other regarding how they change in their school satisfaction, achievement values, and failure anticipation over time. These three constructs were specifically targeted since they have been shown to be associated with interpersonal relationships in school, and are important indicators of academic performance. For example, previous research has shown that changes in students’ school satisfaction are predicted by students’ relationships with peers (e.g., Bayram Özdemir and Stattin 2014) and teachers (e.g., Zullig et al. 2011). Further, school satisfaction has been found to be associated with academic self-efficacy (Huebner and McCullough 2000) and school grades (e.g., Huebner and Gilman 2006). To sum up, developing a good understanding of the possible reasons why adolescents might become dissatisfied with their school or/and develop failure anticipation may be an important step in boosting adolescents’ academic performance and preventing school drop-out.

### The Role of Adolescents’ Individual Characteristics

In their conceptual model of the student-teacher relationship, Pianta and colleagues (Myers and Pianta 2008) indicate that individual features of students, including their gender and ethnic/cultural background, might have an impact on how students perceive the relationship (i.e., students’ cognitive representation of it) and what they need from teachers. Relatedly, all the perceptions and needs may determine the extent to which students are sensitive to the effects of their relationship with teachers. In the present study, it was tested whether the association between teacher-student relationship trajectories and school adjustment outcomes varies according to youth’s gender and immigrant background.

#### Role of gender

The existing literature provides mixed evidence regarding how relationships with teachers might be related to the academic adjustment of girls and boys. Some studies show that girls are at greater risk of developing adjustment problems and showing poor academic performance when they have conflictual relationships with teachers (e.g., McCormick and O'Connor 2015). Likewise, they may benefit more from close and supportive relationships with teachers than boys (e.g., Ewing and Taylor 2009). Relying on the gender socialization hypothesis, these studies argue that girls are expected to follow more traditional roles, such as being compliant and responsible, and relatedly to establish a close relationship with teachers (e.g., Ewing and Taylor 2009; McCormick and O'Connor 2015). Breaking up traditional gender norms (e.g., having less close relationship with teachers) might induce stress in girls, and thereby they might experience school adjustment problems to a greater extent than boys. By contrast, other studies show that boys are more susceptible to the effect of a negative relationship with teachers (Hamre and Pianta 2001). They indicate that boys on average tend to have higher levels of academic and behavioral problems at school than girls. Such vulnerability may make them more prone to the effects of (non) supportive school contexts. Finally, some studies report null findings, suggesting that the quality of relationships with teachers may be equally important for girls and for boys (e.g., Hughes and Cao 2018; Wang and Eccles 2012). Due to the inconclusive findings in the literature, no specific hypothesis was proposed here, and the possible moderating effect of adolescent gender was examined.

#### Role of immigrant status

The existing literature provides mixed evidence on whether the effect of the student-teacher relationship on children’s adjustment varies between immigrant and non-immigrant children. Based on the differential effectiveness model (Muijs et al. 2005), some studies indicate that immigrant youth (particularly first-generation immigrants) have fewer resources and are less familiar with the school system than their native peers (den Brok et al. 2010). Thus, immigrant students rely more on their teachers to adjust and succeed in school. Supportive relationships with teachers may provide immigrant youth with a safe context, which can enhance their sense of belonging, and in turn, their school adaptation. Accordingly, in the Netherlands, perceived close relationships with teachers were found to be more strongly associated with youth interest in subjects taught among non-Dutch youth than among Dutch youth (den Brok et al. 2010). By contrast, other studies report that the link between teacher support and academic outcomes (such as school compliance, school identification, and the value attributed to learning) may not differ between minority and non-minority students (e.g., Wang and Eccles 2012). This suggests that feeling close to, or being supported by, non-parental adult figures in school may be a universal need of young people regardless of their minority/migrant background. Thus, both immigrant and non-immigrant youth may benefit equally from positive relationships with teachers.

## Current Study

In the current study, four-year longitudinal data were used to address three important questions. The first goal was to explore whether adolescents follow different developmental trajectories regarding their perceptions of relationships with teachers during upper-secondary school years (from age 13 to 15). Since the student-teacher relationship is not a static phenomenon (e.g., Bosman et al. 2018; O’Connor and McCartney 2007), it was expected that multiple distinct trajectories would become apparent. Accordingly, adolescents may differ from each other on what they expect from teachers, and relatedly how they perceive their relationship with teachers, over the years. The second goal was to examine whether students on different teacher-relationship trajectories differ from each other regarding how they change in their school satisfaction, achievement values, and failure anticipation over time. Relying on the interpersonal-relationship literature, it was expected that the continuous experience of supportive and fair teacher treatment would be beneficial to adolescents. The final goal was to explore the possible moderating roles of adolescent gender and immigrant background. Given that the current literature offers mixed evidence, no directional hypotheses were proposed regarding these moderation effects.

## Methods

### Participants

The sample for the current study was taken from a longitudinal study, Political Socialization Project (Amnå et al. 2009), which was concerned with identifying the factors that play a role in adolescents’ interest and involvement in civic and political issues during the upper-secondary and high school years. Compulsory education in Sweden starts at age 6 and continues until age 15. Despite some minor differences, compulsory education consists of four stages for most public-sector schools: preschool, low or primary (grades 1–3), middle or lower-secondary (grades 4–6), and high or upper-secondary (grades 7–9). Students start upper-secondary school at age 13, and have a different teacher for each subject (on average a total of 10–15 different teachers). This is similar to many other countries, including the U.S.

The study was conducted in thirteen different schools (10 upper-secondary schools and 3 high schools) from neighborhoods with varying socio-demographic characteristics in a medium-size town in Sweden. The current study focused on the longitudinal sample that included students who were at grade 7 (age 13) during the first year of the study (T1). These students were re-assessed at grade 8 (T2), grade 9 (T3), and after they transferred to high school (T4, age 16). The target sample consisted of 960 students. Of the target sample, 94% participated at T1 (*n* = 904). Among the participating adolescents, 7th grade students who had data on teacher relationships at T1 and also at one of the later assessment points (T2 or T3) were included. The analytic sample for the present study included 829 students (*M*_*age*_ = 13.43, *SD* = 0.55, 51% girls).

A majority of the participating students were born in Sweden (91%) and had parents with a Nordic background (77% of mothers and 76% of fathers). Immigrant background was defined as having a parent who was born outside Sweden or another Nordic country. About 27% of the students were from immigrant families. A majority of the adolescents came from intact families (73%). More than two-thirds of the youth reported that their parents had employment (92% of fathers and 84% of mothers), and perceived their economic conditions as good as or better than people in their class (81%) or in their neighborhood (85%).

### Attrition Analysis

Among the adolescents who participated at T1, 198 (24%) had dropped out by T4. A logistic regression model was estimated to test whether attrition from T1 to T4 was systematic in any way. Attrition (dropout = 1, retention = 0) was regressed on the demographic characteristics of the adolescents (i.e., gender, immigrant status, and perceived socioeconomic status) and all the other study variables (i.e., relationship with teachers, school satisfaction, value attached to achievement, and failure anticipation). The results indicated that only gender (Wald = 7.44, *p* = 0.006, *Exp*(B) = 1.63, 95% CI: 1.15–2.34) and immigrant background (Wald = 4.07, *p* = 0.044, *Exp*(B) = 1.45, 95% CI: 1.01–2.18) significantly predicted attrition (Nagelkerke *R*^*2*^ = 0.04), suggesting that boys and immigrant youth were more likely to drop out. Converting the Exp(B) values for gender and immigrant background into Cohen’s *d* estimates to ease interpretation (Chinn 2000) showed that the effect sizes of both gender (*d* = 0.27) and immigrant background (*d* = 0.20) on attrition were small (Cohen 1988). Thus, it was concluded that attrition had only a minimal effect on the findings.

### Procedure

The data collections were held during regular class hours by trained research assistants. Students were informed about the general aim of the study and the amount of time needed to complete the questionnaire before each data collection. They were also informed that their participation was voluntary, and assured that their responses would be confidential. Only students who were willing to participate and whose parents did not decline their children’s participation took part in the study. Each class received a payment of approximately 100 EUR for their participation. The Regional Research Ethics Committee in Uppsala, Sweden, approved the study and the procedures.

### Measures

#### Fair and supportive relationship with teachers

A three-item scale was used to measure the extent to which adolescents perceived their relationship with teachers as fair and supportive during middle school (Lundberg and Abdelzadeh 2019). The sample items include: “Most of my teachers treat me fairly” (see Appendix A for all scale items). Adolescents responded to each item on a scale ranging from “1” (absolutely agree) to “4” (absolutely disagree). The responses were reverse coded so that a high score refers to a very fair and supportive teacher relationship. The scale had acceptable internal consistency at all three assessment points (0.74, 0.79, and 0.79 at T1, T2, and T3, respectively).

The measurement invariance of this scale over time was tested. The model where the items within each assessment point loaded on its own latent factor fitted the data well, χ^2^(24) = 77.96, *p* < 0.001, CFI = 0.98, RMSEA = 0.05, *p* = 0.373, SRMR = 0.03, suggesting configural invariance of the measures over time. Then, the loadings were constrained to be equal to test the metric invariance. The model fitted the data well, and the overall model fit did not differ from that of the freely estimated model, Δχ^2^(4) = 4.78, *p* = 0.310. Finally, the intercepts were constrained to be equal across time (in addition to loading invariance) to test scalar invariance. The model fit was not statistically significantly different than that of the freely estimated model, Δχ^2^(10) = 10.72, *p* = 0.380. Overall, the findings indicated that the fair-and-supportive-relationship-with-teacher scale had strong measurement invariance over time.

#### School satisfaction

Students were presented with 5 questions to measure the extent to which they were satisfied with and perceived their overall school experiences positively (Kerr and Stattin 2000). The sample item is: “How would you describe the relationship between you and school?” (see Appendix A for all scale items). The response scale ranged from “1” (as best friends) to “5” (as enemies). The responses were reverse coded so that a high score refers to high school satisfaction. Past research has provided evidence for the criterion validity of this scale by reporting significant negative correlations between school satisfaction and perceived academic failure and cutting class (Bayram Özdemir and Stattin 2014). This scale has also been found to have acceptable internal consistency reliability across multiple studies (e.g., Bayram Özdemir and Stattin 2014; Svenson et al. 2011). In the present study, school satisfaction data from the T1 and T4 assessments were used. The inter-item reliability of this scale was acceptable at both T1 and T4 (α = 0.71 and α = 0.72 at T1 and T4, respectively).

#### Achievement values

A revised version of the life value scale was used to measure adolescents’ humanistic, hedonistic, environmental, and achievement values (Stattin and Kerr 2002; Stattin and Kim 2018). In the present study, only the achievement value subscale was used. This subscale captures the extent to which text adolescents value achievement and success. The sample items include: “Always do my best” and “Working hard on school work” (see Appendix A for all scale items). Adolescents reported on the importance of each of these items on a 5-point scale, ranging from “1” (not at all important) to “5” (very important). Previous research has shown only a moderate correlation (*r* = 0.33) between the youth- and parent-reported achievement values of adolescents, which has implications for convergent validity of this construct (Stattin and Kim 2018). In the present study, achievement values data from the T1 and T4 assessments were used. The scale had good internal consistency reliability (α = 0.78 and α = 0.79 at T1 and T4, respectively).

#### Failure anticipation

A four-item scale was used to measure adolescents’ expectations of failure (Nurmi 1993). The sample items are: “I easily become uncertain when I am faced by new tasks” and “Often, I don’t even think there is any point in trying when I am faced by demanding tasks” (see Appendix A for all scale items). The adolescents were asked to report on how much each of the statements applied to them on a four-point scale, ranging from “1” (applies perfectly) to “4” (does not apply at all). The responses were reverse coded so that a high score refers to high failure anticipation. In the present study, failure anticipation data from the T1 and T4 assessments were used. The scale had acceptable internal consistency across different assessment points (α = 0.69 and α = 0.80 at T1 and T4, respectively).

#### Independence of the study variables

The main study variables (i.e., school satisfaction, failure anticipation, achievement values, and perceived teacher support and fairness) are interrelated measures. Thus, a series of confirmatory factor analysis (CFA) models were fitted in order to test whether they refer to empirically distinct constructs. Specifically, the following CFA models were estimated: (1) a single-factor model where all items load onto one latent construct, (2) a four-factor orthogonal model (the four factors are fully independent), and (3) a four-factor oblique model (the four factors are interrelated). The latter two models were nested, which allows comparison of model fit using a chi-square difference test. The single factor model showed a very poor fit, χ^2^(90) = 1300.34, *p* < 0.001, CFI = 0.59, RMSEA = 0.13, *p* < 0.001, SRMR = 0.10. The fit of the orthogonal model was also poor, χ^2^(90) = 703.32, *p* < 0.001, CFI = 0.80, RMSEA = 0.09, *p* < 0.001, SRMR = 0.15. But, the third, oblique, model yielded acceptable fit, χ^2^(84) = 228.28, *p* < 0.001, CFI = 0.95, RMSEA = 0.046, *p* = 0.845, SRMR = 0.042. Comparison between the nested models showed that the oblique model fitted the data significantly better than the orthogonal model, Δχ^2^(6) = 475.04, *p* < 0.001. Overall, these analyses suggested that the study variables were interrelated but independent, with latent factor correlations ranging from −0.16 to 0.55, but they referred to empirically distinct constructs.

### Data Analysis

First, a latent growth curve model in Mplus was estimated (Muthén and Muthén 1998–2010) to examine how the adolescents’ relationships with teachers changed during the upper-secondary school years on average (i.e., from T1 to T3), and also to investigate whether there was significant variation across adolescents regarding how they changed. Next, latent class growth analysis was used to identify whether there were subgroups of children who followed a distinct pattern of change over time (Jung and Wickrama 2008). Latent class growth models (LCGMs) with different cluster solutions were tested, and widely used recommendations to determine the adequate number of trajectory classes were employed (e.g., Jung andand Wickrama 2008; Nylund et al. 2007). Specifically, the model with the lowest on the Bayesian Information Criterion (BIC, Kass and Raftery 1995), and with the highest classification accuracy (i.e., entropy, Muthén 2004) was chosen. Further, Lo-Mendell-Rubin adjusted likelihood ratio tests were conducted (LMR-LRT, Lo et al. 2001) to examine whether there were significant improvements in model fit from the *n-1* trajectory model to the *n* trajectory model. A non-significant difference would suggest that the *n-1* class solution is preferable. After identification of the final trajectory classes, latent-change models were estimated to examine how the teacher relationship trajectories were linked to the changes in adolescents’ school satisfaction, achievement values, and failure anticipation.

The students were clustered in different schools and classrooms, which violates the assumption of independence of observations. The intra-class correlations (ICCs) for all study variables were estimated to identify how much of the observed variation was due to differences across the schools or classrooms. The ICC estimates suggested that, on average, 3% of the observed variations in the study variables were due to differences across schools, whereas 5% of these variations were due to differences across classrooms. Thus, the TYPE = COMPLEX command in Mplus was used to control for clustering effects in all models, so as to obtain corrected standard error estimates and unbiased test statistics, and to eliminate the possibility of a Type-I error. In order to handle missing data, the expectation-maximization (EM) method was used before estimating the regression models. The EM method has been shown to be more effective than traditional missing data approaches (e.g., listwise deletion, pairwise deletion, or mean imputation) in eliminating Type-II errors, so as not to underestimate correlations and standardized regression coefficients (Acock 2005; Kline 2011). Nagin (2005) recommended having 300–500 cases for adequate power to detect identifiable trajectories in LCGM analysis. The analytic sample of the current study is substantially larger than the recommended sample size, so sample size may not limit the power to detect identifiable trajectories.

## Results

### Descriptive Statistics and Preliminary Analyses

Means, standard deviations, and correlations among the study variables are presented in Table 1. All the study variables were associated with each other in the expected directions. Specifically, a fair and supportive teacher relationship was positively correlated with school satisfaction and achievement value, and negatively correlated with failure anticipation. Swedish and immigrant youth did not significantly differ from each other on how they perceived their relationships with teachers. Also, except at the T1 assessment, there was no significant difference between girls and boys on how they perceived their relationships with teachers.

**Table 1:** Correlations, means, and standard deviations for the study variables

	1	2	3	4	5	6	7	8	9	10	11
1. Gender	–										
2. Immigrant status	–0.04	–									
3. Relationship with teachers—T1	0.11**	–0.01	–								
4. Relationship with teachers—T2	0.03	0.02	0.47***	–							
5. Relationship with teachers—T3	0.01	–0.01	0.35***	0.50***	–						
6. School satisfaction—T1	–0.03	0.02	0.48***	0.36**	0.30***	–					
7. School satisfaction—T4	0.01	–0.06	0.27***	0.29***	0.35***	0.39***	–				
8. Failure anticipation—T1	–0.13***	0.08*	–0.21***	–0.14***	–0.13**	–0.34***	–0.24***	–			
9. Failure anticipation—T4	–0.25***	0.04	–0.16***	–0.20***	–0.19***	–0.25***	–0.45***	00.44***	–		
10. Achievement value—T1	–0.06	0.16^***^	0.25***	0.22***	0.10**	0.40***	0.16***	–0.11**	–0.04	–	
11. Achievement value—T4	–0.08*	0.16***	0.11**	0.19***	0.20***	0.28***	0.42***	–0.12**	–0.26***	0.39***	–
*M*	–	–	3.11	3.08	3.09	3.74	3.80	2.22	2.00	4.43	4.25
*SD*	–	–	0.57	0.60	0.59	0.65	0.62	0.60	0.64	0.56	0.67

Gender was coded as: “1” boy and “0” girl. Immigrant status was coded as “1” immigrant and “0” Swedish

*p < 0.05; **p < 0.01; ***p < 0.01

### Do Adolescents’ Relationships with Teachers Change over Time?

A latent growth curve model was estimated to examine whether the adolescents’ relationships with their teachers changed during the upper-secondary school years. The model fitted the data well, χ^2^(1) = 0.25, *p* = 0.61, CFI = 1.00, RMSEA = 0.00, 950% CI for RMSEA 0.00–0.07, SRMR = 0.006. The mean of the slope was not statistically significant, which indicates that, on average, there was no significant change in adolescents’ relationships with their teachers over three years. However, the variances of both intercept and slope were statistically significant. That is, there were significant inter-individual differences in how adolescents viewed their teachers at T1 and how their perceptions changed over time. These findings suggest that there might be unique clusters of adolescents who follow different growth trajectories. It was also tested whether the adolescents reported different levels of quality of the perceived teacher-student relationship, or showed different rates of change over time, based on their gender and immigrant background. These two variables were included as predictors in the LGM model, and boys reported slightly but significantly higher levels of positive relationship with teachers at T1, *β* = 0.15, *p* = 0.002. There were no differences in the perceived teacher-student relationship based on immigrant background. Further, neither gender nor immigrant background was related to the rate of change in the teacher-student relationship over time.

### Trajectories of Adolescents’ Relationship with Teachers

LCGMs with different cluster solutions were estimated to examine whether the adolescents followed different trajectories in their relationships with teachers. The fit statistics for all class solutions are presented in Table 2. As shown in the table, the two-, three-, and four-class solutions showed improvements in BIC and Entropy. The results of the LMR-LRT were statistically significant for the two-class and three-class solutions. However, the LMR-LRT was not statistically significant for the four-class solution, which indicated that moving from a three-class to a four-class solution did not improve model fit-to-data. Thus, the three-class solution was retained (as it fitted the data best). In addition, the model parameters for the three-class solution were replicated using the OPTSEED command, which suggests that the cluster solutions were robust.

**Table 2:** Latent class growth model fit indices for a fair and supportive relationship with teachers

Class	AIC	BIC	Entropy	LMR-LRT
1 Class	4157.40	4180.99	–	–
2 Class	3882.05	3919.82	0.68	0.02
3 Class	3744.08	3796.00	0.80	0.03
4 Class	3659.49	3725.58	0.85	0.57

**Fig. 1:**
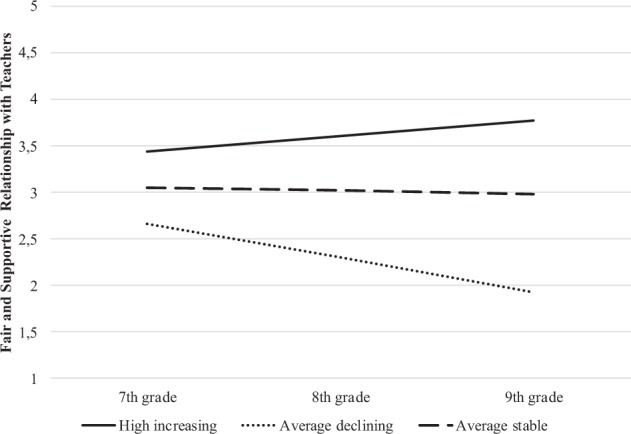
Teacher relationship trajectories

As shown in Table 3, the first class (*high-increasing trajectory*) represented 24% of the sample. The adolescents who were on this trajectory reported a high level of fair and supportive teacher-relationships at T1, and continued to increase in their positive views over time. About 66% of the adolescents were in the second class (*average-stable trajectory)*. They reported an average level of positive relationships with teachers at T1 and did not change significantly over the three years. The final class (*average-declining trajectory*) comprised adolescents who reported an average level of positive relationships with teachers at T1 (10% of the sample); however, they showed a decline in their positive views towards teachers over time. The three latent clusters on youth’s perceptions of relationships with teachers at all three waves were compared. The results suggested that the three clusters differed significantly in their perceptions of relationships with teachers at T1, *F*(2, 826) = 96.44, *p* < 0.001, at T2, *F*(2, 783) = 226.04, *p* < 0.001, and at T3, *F*(2, 726) = 1376.96, *p* < 0.001. Overall, two-thirds of the adolescents displayed a normative trend, *average-stable*, in their perceived relationship with teachers. On the other hand, one-fifth of the adolescents (*high-increasing*) improved in their already positive perceptions, while one in ten of the adolescents (*average-declining*) declined in their perceived relationship with teachers (i.e., average-declining). The estimated mean trends for the three trajectories are shown in Fig. 1.

**Table 3:** Parameter estimates for each trajectory class

Trajectory class			
Estimates	High increasing (*n* = 194)	Average stable (*n* = 550)	Average declining (*n* = 85)
Mean intercept	3.45***	3.05***	2.67***
Mean slope	0.16***	–0.03	–0.37***

***p < 0.001

### The Link between Adolescent Individual Characteristics and Teacher Relationship Trajectories

A multinomial logistic regression analysis was conducted to test whether the adolescents’ demographic characteristics, including their gender and immigrant status, predicted classification into one of the latent trajectory classes. Neither adolescent gender nor immigrant status significantly predicted class membership.

### Teacher Relationship Trajectories and Changes in Adolescents’ School Adjustment

Regression models were estimated to examine whether students on different teacher-relationship trajectories differed from each other on how they changed in their school satisfaction, achievement values, and failure anticipation over time (i.e., from T1 to T4). In all models, adolescents on an average-stable trajectory were treated as the reference group. Further, T1 assessments of the outcome variables were controlled for in order to estimate the change scores.

#### School satisfaction

Adolescents on a high-increasing trajectory reported greater school satisfaction over time than those in the average-stable group. Further, adolescents in the average-declining group reported lower school satisfaction over time than those in the average-stable group. The adolescents’ gender and immigrant status did not significantly predict change over time in school satisfaction (see Table 4). It was also explored whether there were moderating effects of adolescents’ gender and immigrant status. In order to estimate these effects, interaction terms were created between immigrant status (or gender) and dummy variables. None of the interaction effects were statistically significant, suggesting that immigrant and Swedish adolescents, and also girls and boys, on different trajectories changed similarly in their school satisfaction over time.

**Table 4:** Teacher relationship trajectories and change in adolescents’ adjustment

	Outcome variables		
	School satisfaction at T4	Achievement value at T4	Failure anticipation at T4
Gender	0.04	–0.04	–0.16***
Immigrant status	–0.06	0.07	0.01
T1 assessment of outcome variable	0.37***	0.42***	0.44
Average decreasing trajectory^a^	–0.15***	–0.06**	0.06*
High increasing trajectory^a^	0.15***	0.11***	–0.12**
*R*^*2*^	0.25***	0.22***	0.28***

Gender was coded as: “1” boy and “0” girl. Immigrant status was coded as “1” immigrant and “0” Swedish

^a^Average stable trajectory was defined as reference category

*p < 0.05; **p < 0.01; ***p < 0.001

#### Achievement value

Relative to adolescents on an average-stable trajectory, adolescents on a high-increasing trajectory reported higher achievement values over time. By contrast, adolescents in the average-declining group valued achievement less over time. The main effects of adolescent’s gender and immigrant status were not statistically significant (see Table 4). There were no significant interaction effects.

#### Failure anticipation

Adolescents on a high-increasing trajectory reported lower failure anticipation over time than those in the average-stable group. Further, the adolescents in the average-declining group reported higher failure anticipation over time than those in the average-stable group. There was a significant main effect of adolescent gender, such that girls showed higher failure anticipation over time than boys (see Table 4). None of the interaction effects were statistically significant, suggesting that adolescents on different trajectories changed similarly in their failure anticipation over time, regardless of their gender or immigrant status.

## Discussion

Adolescents interact with their teachers on a daily basis, and their experiences with teachers may change over the years. Some adolescents may maintain their positive relationship with teachers whereas as others’ relationship may worsen, and the pattern of the change in adolescent-teacher relationship may have implications for adolescent’s adjustment. However, the adolescent-teacher relationship has generally been measured at a single point in time, and is assumed to be static during adolescence (except in a few studies; e.g., Reddy et al. 2003). This approach does not provide any information regarding how relationships with teachers evolve over time, and how possible changes are related to young people’s adjustment. The present study extends the body of research by examining trajectories in student-teacher relationship quality, and their associations with young people’s adjustment.

The findings suggest that not all adolescents change in their relationship with teachers in the same direction. Three distinct subgroups were identified, which are somewhat similar in trajectory to those identified in a previous study focusing on the early childhood period (Miller-Lewis et al. 2014). Specifically, about a quarter of the adolescents (24%) perceived their teachers as supportive and fair when they were 13 years-old, and continued to increase in their positive views over time. More than half of the young people (66%) started off with average levels of positive relationships with teachers, and remained the same up until they were 15 years-old. A small but significant percentage of the adolescents (10%) had a somewhat positive relationship with their teachers at the beginning of the lower-secondary years, but their relationship got worse over time. Together, these findings suggest that a majority of young people perceive their relationship with teachers as supportive and fair during adolescence, and that the relationship is either maintained or gets better over time. However, a small group of adolescents perceive their teachers as less supportive and fair as they get older, which is a cause for concern.

Consistent with the expectation and previous research (e.g., Bosman et al. 2018; O’Connor and McCartney 2007), the results showed that adolescents on a high-increasing trajectory had better school adjustment than those on the average-stable and average-declining trajectories. More importantly, adolescents in the average-declining group reported a decrease in school satisfaction and achievement value, and an increase in failure anticipation, relative to those on the average-stable trajectory. In line with previously presented theoretical arguments, these findings suggest that forming a positive relationship with teachers, and, importantly, maintaining it over time may convey the message to adolescents that they are valued and cared for in the school setting. Such perceptions may help young people build a sense of belonging in and connection to the school, and in turn, internalize academic goals. By contrast, when adolescents’ positive views about teachers change drastically (e.g., after receiving less support from teachers or being exposed to unfair teacher treatment), it is possible that they become insecure and distressed at school, and in turn, experience various forms of adjustment difficulties. In fact, a recent study (Bayram Özdemir et al. under review) shows that the experience of unfair teacher treatment takes its toll on the psychological well-being of Swedish youth, and in turn, makes them less engaged in class activities or even avoidant of school over time. In sum, the findings indicate that the student-teacher relationship is not static during adolescence, and that the continuous experience of supportive and fair teacher treatment over time (rather than that of a positive relationship at a certain moment in time) facilitates adolescents’ school adjustment.

In the present study, we also examined how the relationship with teachers is related to the adjustment of girls and boys separately. The results suggest that adolescents on different trajectories change similarly in their school satisfaction, achievement value, and failure anticipation over time, regardless of gender. This finding, however, is contrary to the results of studies relying on the gender socialization hypothesis, which indicate that forming a close relationship is more important for girls, and that girls may benefit more from having a close and supportive relationship with teachers (e.g., Ewing and Taylor 2009). Nevertheless, the finding is in line with the results of recent studies (e.g., Hughes and Cao 2018; Wang and Eccles 2012), which suggest that the quality of relationships with teachers becomes equally important for girls and boys during adolescence. It is possible that gender-specific socialization patterns in student-teacher relationships may be more prominent during early (Ewing and Taylor 2009) and later childhood (McCormick and O'Connor 2015). Relatedly, girls may be more sensitive to (un)supportive relationships with teachers than boys during these developmental periods. However, gender-specific socialization patterns in the student-teacher relationship may become less nuanced during adolescence, and thus the effect of the quality of the relationship with teachers on young people’s adjustment may not differ between boys and girls.

In line with previous research (e.g., Wang and Eccles 2012), we found that over time changes in the student-teacher relationship have a similar impact on the school adjustment of immigrant and native adolescents. This finding supports interpersonal theories (e.g., Baumeister and Leary 1995) and equity theory (Adams 1965), which postulate that being cared for and valued, and having a sense of justice in a social setting, are essential psychological needs of every young individual, regardless of their ethnic, cultural, or immigrant background. When these needs are fulfilled, adolescents are more likely to build a sense of belonging and connection, and thereby to function well.

Despite its important contributions to the literature, several limitations of the present research need to be acknowledged. First, the present study focused on subjective school outcomes (e.g., school satisfaction and achievement values), and thus the findings are rather limited in their explanation of the extent to which changes over time in the student-teacher relationship determine adolescents’ academic performance over time. Thus, future research focusing on objective school outcomes (e.g., grade point average, GPA, or other educational attainments) would be useful fully to understand how growth trajectories in student-teacher relationships are linked to changes over time in school adjustment and functioning during adolescence. Second, although some demographic variables were included as covariates (e.g., gender and immigrant status) and controlled for clustering effects, there may have been unmeasured factors that would have had an impact on the results. Thus, accounting for the effects of possible confounders (e.g., quality of the relationship with peers at school) in future research would help us to draw more robust conclusions. Third, in line with the previous literature (e.g., Engels et al. 2016; Wang and Holcombe 2010), adolescents were asked to report on their overall relationship with teachers at school, rather than their relationship with a single or specific teacher. This measurement approach was taken because young people are in contact with multiple teachers during upper-secondary school, and it was simply not feasible to ask them to report on their relationship with every single teacher. Even though this measurement approach gives us a picture of how adolescents perceive their relationship with teachers overall, it should be acknowledged that this approach may lead to a lack of specificity in measurement. In turn, it limits the ability, for example, to test whether certain characteristics of teachers (e.g., their gender and cultural background) play a role in the association between the teacher-student relationship and adolescents’ school adjustment, or to examine the effect of different teacher relationship patterns (e.g., having one fair and supportive teacher but others who are not versus having teachers who are all fair and supportive). Finally, the present study provides us with valuable information about how the student-teacher relationship changes during adolescence and its possible effects on youth. However, it is rather limited with regard to providing information about the precursors of this change. Identifying the possible underlying mechanisms that lead young people to follow different trajectories would be informative for programs aiming to promote adolescents’ school adjustment.

## Conclusion

The present study sheds light on how the student-teacher relationship evolves during adolescence. The findings clearly suggest that the relationship is dynamic, and that adolescents follow different trajectories over time. In line with the conceptual arguments in the interpersonal-relationship literature, the findings indicate that the continuous experience of supportive and fair teacher treatment (rather than the experience of a positive relationship at a certain point in time) is beneficial for the adjustment of youth in a school setting. Importantly, the findings were the same for both boys and girls, and for both immigrant and native students, which suggests that being cared for and experiencing equal treatment are essential psychological needs of every young individual. Systematic efforts in a school context to improve and maintain fair and supportive relationships between students and teachers might help promote the school adjustment of all students, regardless of gender and of immigrant or native background.

## Appendix A: Study Constructs and Scale Items

**Table:**

Construct	Scale items	Response scale
1. Fair and supportive relationship with teachers	1. Most of my teachers treat me fairly.	1 = Absolutely agree, 4 = Absolutely disagree
	2. Most of my teachers listen to what I have to say.	
	3. Most teachers are keen that their students feel good.	
2. School satisfaction	1. How do you like it in school?	1 = Very good, 5 = Very bad
	2. Do you do the best that you can in school?	1 = Usually, 5 = Almost never
	3. Do you feel like you are forced to go to school?	1 = Most often, 5 = Almost never
	4. How would you describe the relationship between you and school?	1 = As best friends, 5 = As enemies
	5. Are you satisfied with how it’s going for you in school?	1 = Usually, 5 = Almost never
3. Achievement values	1. Always doing my best	1 = Not at all important, 5 = Very important
	2. Working hard on school work	
	3. Studying so that I get good grades	
	4. Learning new things	
4. Failure anticipation	1. I easily become uncertain when I am faced with new tasks	1 = Applies perfectly, 4 = Does not apply at all
	2. I don’t really have any confidence in my ability to cope with difficult tasks	
	3. Often, I don’t even think there is any point in trying when I am faced with demanding tasks	
	4. Just the feeling that I find it hard to cope with things means that I don’t succeed as well as I should be able to	
